# Power Equipment Fault Diagnosis Method Based on Energy Spectrogram and Deep Learning

**DOI:** 10.3390/s22197330

**Published:** 2022-09-27

**Authors:** Yiyang Liu, Fei Li, Qingbo Guan, Yang Zhao, Shuaihua Yan

**Affiliations:** 1 Key Laboratory of Networked Control Systems, Chinese Academy of Sciences, Shenyang 110016, China; 2 Shenyang Institute of Automation, Chinese Academy of Sciences, Shenyang 110016, China; 3 Institutes for Robotics and Intelligent Manufacturing, Chinese Academy of Sciences, Shenyang 110169, China; 4 College of Information Science and Engineering, Northeastern University, Shenyang 110819, China; 5 Shenzhen TCL New Technology Company, Shenzhen 518000, China; 6 School of Automation and Electrical Engineering, Shenyang Ligong University, Shenyang 110159, China; 7 School of Computer Science and Technology, University of Chinese Academy of Sciences, Beijing 100049, China

**Keywords:** smart grid, power equipment, fault detection, energy spectrum feature map, Dense Residual Networks, transfer learning, channel domain attention

## Abstract

With the development of industrial manufacturing intelligence, the role of rotating machinery in industrial production and life is more and more important. Aiming at the problems of the complex and changeable working environment of rolling bearings and limited computing ability, fault feature information cannot be effectively extracted, and the current deep learning model is difficult to be compatible with lightweight and high efficiency. Therefore, this paper proposes a fault detection method for power equipment based on an energy spectrum diagram and deep learning. Firstly, a novel two-dimensional time-frequency feature representation method and energy spectrum feature map based on wavelet packet transform is proposed, and an energy spectrum feature map dataset is made for subsequent diagnosis. This method can realize multi-resolution analysis, fully extract the feature information contained in the fault signal, and accelerate the convergence of the subsequent diagnosis model. Secondly, a lightweight residual dense convolutional neural network model (LR-DenseNet) is proposed. This model combines the advantages of residual learning and a dense connection, and can not only extract deep features more easily, but can also effectively use shallow features. Then, based on the lightweight residual dense convolutional neural network model, an LR-DenseSENet model is proposed. By introducing the transfer learning strategy and adding the channel domain, an attention mechanism is added to the channel feature fusion layer, with the accuracy of detection up to 99.4%, and the amount of parameter calculation greatly reduced to one-fifth of that of VGG. Finally, through an experimental analysis, it is verified that the fault detection model designed in this paper based on the combination of an energy spectrum feature map and LR-DenseSENet achieves a satisfactory detection effect.

## 1. Introduction

With the development of industrial manufacturing intelligence, the role of rotating machinery in industrial production and life is more and more important. As the most common rotating machinery and equipment, rolling bearings work in complex and changeable harsh environments for a long time, so it is inevitable that there will be some damage, if not timely treatment. Because the failures will cause huge losses to industrial production, it is necessary to carry out scientific and efficient fault diagnosis research on rolling bearings [1].

In recent years, people have proposed many fault detection methods for rolling bearings based on vibration signal analysis. Signal feature extraction and intelligent diagnosis algorithms are two core steps of bearing fault detection research that play a decisive role in the final effect of fault detection.

Most scholars use the time-frequency domain analysis method to extract fault signal features. Time-frequency domain analysis methods mainly make use of the joint distribution of time domain information and frequency domain information to conduct correlation analysis of signals, among which wavelet transform [2], singular value decomposition [3], short-time Fourier transform [4], wavelet packet transform [5], ensemble empirical mode decomposition [6], and other time-frequency analysis methods are widely used in the field of fault detection. However, although the abovementioned methods claim certain achievements, there are still some common limitations: the first is the feature extraction conducted mainly through technical personnel artificial extraction, which relies on the expert’s experience and lack of generalization, especially when the power equipment is more complex or in operation mode, etc., making the traditional feature extraction method difficult to effectively extract the fault characteristic information; Secondly, most of the current feature extraction methods are equipped with shallow classification models [7,8,9], and the simple architecture of these models limits the nonlinear processing of fault feature information. Therefore, with the rapid development of industrial big data, it is necessary to study feature extraction methods with more adaptability and generalization and classification models with better classification performance.

In recent years, with the increasing maturity of artificial intelligence technology, some deep learning models that can achieve adaptive extraction of vibration signal fault features are gradually occupying the mainstream position, such as convolutional neural network [10], deep belief network [11], self-coding network [12], recurrent neural network [13], etc. They have been applied to the fault detection field and achieved good results. However, traditional signal analysis combined with shallow learning network for fault detection relies on the professional knowledge of researchers, and has strong uncertainty in feature extraction, which has a great impact on detection performance. The fault detection algorithm based on deep learning correlation architecture [14] combines feature extraction with fault identification and classification by virtue of its adaptive extraction ability of fault features, which significantly improves the efficiency and generalization of fault detection.

However, with the deepening of layers, the corresponding problems of deep learning network architecture begin to appear. The over-fitting caused by too many deep layers, the disappearance of gradients, and the increase of parameter calculation amount on the hardware requirements are some problems at the present stage. Therefore, to find a more efficient intelligent fault diagnosis model is the key work of current research.

Firstly, in view of the nonlinearity and instability of rolling bearing fault signals in power equipment under complex working conditions, most of the existing mainstream diagnostic models are unable to process one-dimensional time-domain vibration signals effectively and directly. In this paper, a novel two-dimensional time-frequency feature representation method and energy spectrum feature map is proposed. The branch nodes were obtained by wavelet packet decomposition, the energy information of each node was calculated and normalized to obtain the two-dimensional energy spectrum feature map, and the energy spectrum feature map dataset was made for subsequent detection. This method can realize multi-resolution analysis, realize more detailed analysis of the signal, and fully extract the feature information contained in the fault signal to accelerate the convergence of the subsequent detection model and improve the accuracy of the fault detection model.

Secondly, in this paper, considering that the rolling bearing working environment in power equipment is complicated, the calculation ability is limited, lightweight, and efficient. In view of the current deep learning model, compatibility is a problem, so the characteristics of the two-dimensional power spectrum matrix, combined with the residual deep connection, make it easier to extract features, and the characteristics of the dense connection make effective use of shallow characteristics. In this paper, a novel Lightweight Dense Residual Network model (LR-DenseNet) based on residual connection and dense convolution is proposed. It strengthens the information flow between each layer of the deep network model, makes the transfer of features and gradients more effective, and alleviates the problem of vanishing gradients. At the same time, based on maintaining superior diagnostic accuracy, it greatly reduces the number of parameters and computational complexity of the model, and still has a good diagnostic effect under a variable load environment.

Finally, considering that the training time of the LR-DenseNet model is long and the diagnostic performance needs to be further optimized, a Lightweight Dense Residual Network based on channel attention (LR-DenseSENet, Lightweight ResNet) traffic is dense and the Connected Squeeze-and Excitation Convolutional Neural Network is proposed. Firstly, transfer learning strategy is introduced to shorten the training time by cross-domain transfer learning. At the same time, an improved feature fusion layer based on the channel attention module is proposed to improve the classification accuracy of the model, and greatly improve the reliability and robustness of the model.

## 2. Related Work

With the development of artificial intelligence technology, a lot of research has been done on the fault diagnosis of rotating machinery. To sum up, the research of fault diagnosis methods mainly goes through several stages. The first stage involves human perception. The second involves expert experiences to subjective judgment of fault condition, where the diagnosis process is relatively simple, but the influence of subjective factors leads to the failure of criteria accuracy. At the same time, the methods are restricted to those with professional skills, as the knowledge and cannot be mastered by many, leading to low commonality; With hardware such as sensors, signal analysis, and data processing technology, combined with the rapid advance of the second stage, some scholars use hardware such as sensor acquisition of rolling bearing vibration data. Based on this information, using vibration signal analysis technology for data analysis, and using the results obtained from the analysis of diagnosis, allows for the concept of diagnosis that is in use today. Yuwono et al. [15] put forward a kind of intelligent fault diagnosis methods, and was the first to use wavelet transform and cepstrum filtering completed work to extract fault features, and then based on the swarm optimization algorithm with the hidden Markov model, it was improved, and has carried on in the fault diagnosis on which this work is based. In the case of western reserve data sets, accuracy reached 97.32%. Subsequently, with the continuous development of computer software and hardware technology and the continuous improvement of artificial intelligence technology, scholars began to combine deep learning-related algorithms with signal analysis technology for fault diagnosis. Tamilselvan et al. [16] used multiple sensors to fuse the monitored data. Then, based on DBN, multi-sensor fault health diagnosis is realized.

Among them, the second and third stages of the research experience of rotating machinery fault diagnosis have the same point, which is the two core steps of signal feature extraction and intelligent diagnosis algorithm research. These two steps play a decisive role in the final effect of fault diagnosis. Next, this paper will further introduce its research status.

Fault feature extraction can be divided into three types according to the different perspectives of signal analysis: fault feature extraction based on time domain signals [17], fault feature extraction based on frequency domain signals [18], and fault feature extraction based on time and frequency domain signals. Many scholars use time-frequency domain analysis method to extract fault signals. Different from STFT [19] and EMD [20], wavelet packet decomposition retains the effective local frequency analysis of wavelet decomposition, and further decomposes different frequency ranges to achieve multi-resolution analysis of signals. Li [21] realized the diagnosis and classification of bearing faults based on wavelet packet decomposition and multi-fault classifiers composed of multiple support vector mechanisms. Wu [22] combined wavelet packet decomposition and high order cumulant to effectively extract fault features and use principal component analysis algorithm for dimensionality reduction, thus achieving effective identification of rolling bearing fault types. However, the fault signals of rolling bearings have the characteristics of being non-stationary and unstable, leading to traditional time-frequency domain methods that cannot fully and effectively characterize the characteristic information of vibration signals.

After extracting the characteristic information of vibration signal by analyzing it, it is necessary to classify it for fault detection, which is the key to the whole fault detection research [23]. The fault detection model is classified based on traditional signal analysis and shallow machine learning algorithm, and the algorithms mainly used include artificial neural network and support vector machine [24,25]. Tabrizi [26] obtained eigenvalue vectors of fault signals by means of ensemble empirical mode decomposition and realized further diagnosis and classification of faults by means of support vector machines. Hu [27] obtained feature vector matrix by singular spectrum decomposition and combined a bilevel support vector machine for fault classification. Tan [28] used wavelet packet decomposition to obtain characteristic information of vibration signals, and then carried out diagnostic classification of feature information based on the k-nearest neighbor algorithm.

Convolutional neural network (CNN) and VGGNet, AlexNet, residual convolutional neural network, and dense convolutional neural network are the most widely used algorithms in deep learning, which can extract the features of such irregular time series well. Li et al. [29] designed a multi-scale multi-sensor feature fusion convolutional neural network (MSMFCNN), which fused the rich information provided by multiple sensors and conducted fault diagnosis based on CNN, achieving good diagnosis results. Xiong et al. [30] proposed an enhanced deep residual network with multilevel correlation information for fault diagnosis of rotating machinery, which is used to process the feature information obtained by wavelet packet transformation. Shi et al. [31] proposed a fault diagnosis method based on IMFs and WDenseNets, in which the components of vibration signals obtained through empirical mode decomposition were weighted and input into WDenseNets for fault identification and classification [32]. 

In summary, traditional signal analysis combined with shallow learning network for fault diagnosis relies on the professional knowledge of researchers, and has strong un-certainty in feature extractions, which has a great impact on diagnosis performance. The fault diagnosis algorithm based on deep learning correlation architecture combines feature extraction with fault identification and classification by virtue of its adaptive ex-traction ability of fault features, which significantly improves the efficiency and generalization of fault diagnosis [33]. However, with the deepening of layers, the corresponding problems of deep learning network architecture begin to appear. The over-fitting caused by too many deep layers, the disappearance of gradients, and the increase of parameter calculation amount on the hardware requirements are some problems at the present stage. Therefore, to find a more efficient intelligent fault diagnosis model is the key work of current research.

Table 1 shows the different attributes of deep learning technology fault diagnosis.

## 3. Methods Proposed in this Paper

**Figure 1:**
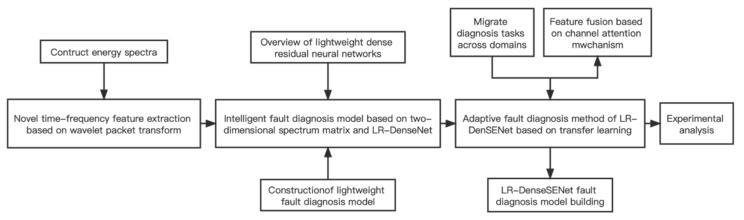
Modular flow chart.

In view of the above limitations, the main research objectives of this topic are: based on the design, to effectively extract fault feature information and use it in the form of two-dimensional data as the subsequent network input feature analysis method. At the same time, the intention was to design a lightweight deep fault diagnosis network model, so that it can ensure the diagnosis effect and reduce the parameter calculation involved in model training. The overall framework of this paper is shown in Figure 1.

### 3.1. Two-Dimensional Time-Frequency Feature Representation Based on Wavelet Packet Transform

The wavelet packet nodes obtained after wavelet packet transformation contain a large amount of information about vibration signals, and the wavelet packet nodes [34] have stronger energy stability in comparison. Based on this, a novel two-dimensional time-frequency feature expression method based on wavelet packet time-frequency transformation is proposed in this paper. This method can fully extract the feature information contained in the fault signal and improve the accuracy of the subsequent fault detection model while realizing multi-resolution analysis.

The calculation process of energy information of branch nodes obtained by wavelet packet transformation is as follows:

(1) Based on the original vibration data signals f(t), N layer wavelet packet decomposition and signal reconstruction are carried out to obtain a total of $2^{N}$ branch reconstruction signals.
(2) The corresponding branch band energy $E_{i,j}$ can be obtained through calculation. The calculation expression of the total energy value contained in the original vibration signal is shown in Equation (1).

(1) $$E=\int_{-\infty }^{+\infty }f^{2}\left(t\right)dt=\overset{2^{j}}{\underset{m=1}{\sum }}\overset{2^{j}}{\underset{n=1}{\sum }}\int_{-\infty }^{+\infty }f_{j,m}\left(t\right)f_{j,n}\left(t\right)dt=\overset{2^{j}-1}{\underset{i=0}{\sum }}E_{i,j}\;$$

where i=0,1,2,⋯,n represent the layers of wavelet packet decomposition, and $j=0,1,2,\cdots ,2^{n}$ are the index of different branch nodes.

B is defined as the decomposition coefficient, $S_{i,j}$ is the branching signal, and $E_{i,j}$ represents the energy value contained in the reconstructed signal $S_{i,j}$. $B_{i,j}(n)$ is the corresponding wavelet packet coefficient, and the frequency band energy information contained in the *j*-th node of the *i*-th layer is shown in Equation (2), where N represents the number of wavelet packet branch nodes contained in the subspace.

(2) $$E_{i,j}=\int \left|S_{i,j}\left(t\right)\right|dt=\overset{N}{\underset{n=1}{\sum }}{\left[B_{i,j}\left(n\right)\right]}^{2}$$

We obtained the energy information contained in specific branch nodes through the previous steps, and then normalized the total energy of the original vibration signal to obtain the corresponding relative energy information of a branch frequency band, as shown in Equation (3).

(3) $$e_{i,j}=\frac{E_{i,j}}{E}=\frac{E_{i,j}}{\sum_{n=1}^{2^{i}}E_{i,j}}\;$$

where $e_{i,j}$ represents the energy value of the corresponding node after normalization. E is the total energy. After the energy information of all nodes is obtained, the energy spectrum feature vector F is constructed, and its expression is shown in Equation (4).

(4) $$F=\left[e_{i,0},e_{i,1},\ldots ,e_{i,2^{i}-1}\right]$$

On this basis, the energy spectrum matrix is constructed, as shown in Equation (5).

(5) $$F=\left[\begin{array}{ccc} e_{i,1} & \begin{array}{cc} e_{i,2} & \cdots \end{array} & e_{i,k}\\ \begin{array}{c} e_{i,k+1}\\ \vdots \end{array} & \begin{array}{cc} \begin{array}{c} e_{i,k+2}\\ \vdots \end{array} & \begin{array}{c} \ldots \\ \vdots \end{array} \end{array} & \begin{array}{c} e_{i,2k}\\ \vdots \end{array}\\ e_{i,m} & \begin{array}{cc} e_{i,m+1} & \ldots \end{array} & e_{i,2^{i}} \end{array}\right]\;$$

Experimental samples for our sample were built in accordance with the rules of 8-layer wavelet packet decomposition and reconstruction: 256 branch nodes, with corresponding wavelet packet coefficient to calculate the corresponding energy information, are obtained, and, finally, all the node energy information is evaluated for a building energy spectrum characteristic figure, and as the subsequent network model input. 

The above notation descriptions are shown in Table 2.

### 3.2. Intelligent Fault Detection Model of LR-DenseNet

The LR-DenseNet intelligent fault detection model proposed in this paper is a new lightweight CNN architecture that integrates residual learning and dense connection ideas. It strengthens the information flow between each layer of the deep network model, makes the transfer of features and gradients more effective, and alleviates the problem of vanishing gradients. At the same time, based on maintaining superior diagnostic accuracy, the number of parameters and computational complexity of the model are greatly reduced.

**Figure 2:**
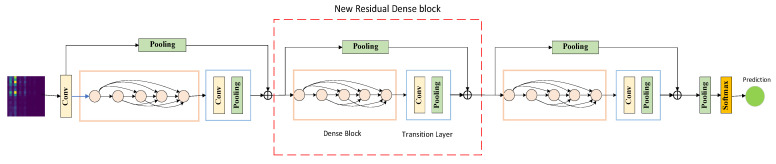
LR-DenseNet structure diagram.

The LR-DenseNet proposed in this paper mainly consists of three parts: a shallow spectrum image feature extraction module, a Local Residual Dense Block (LRDB), and a Dense fault feature abstraction module. The specific network structure is shown in Figure 2. Firstly, the shallow module extracts the features of the input data. Subsequently, each local dense residual module fuses the input shallow level feature information with the features obtained after processing the dense convolution layer, and the feature fusion used here is realized in the form of a short connection. Finally, the operation of feature abstraction and simplification should be added in the process of feature extraction, and the mean pooling layer should be set after the LRDB module, which can further abstract and process the features after fusion. Only the abstract features useful for classification should be retained, which is suitable for complex data structures such as energy spectrum images.

(1) Shallow feature extraction

The input energy spectrum characteristic map is processed, and the shallow level information of the image is extracted through a convolution operation, and it is used as the input of the subsequent LRDB module. At the same time, 64 3 × 3 convolution kernels were used to complete feature extraction, and local information of the spectrum was extracted accurately and effectively. The ReLU activation function is used for nonlinear transformation.

(2) Local Residual Dense Block (LRDB)

**Figure 3:**
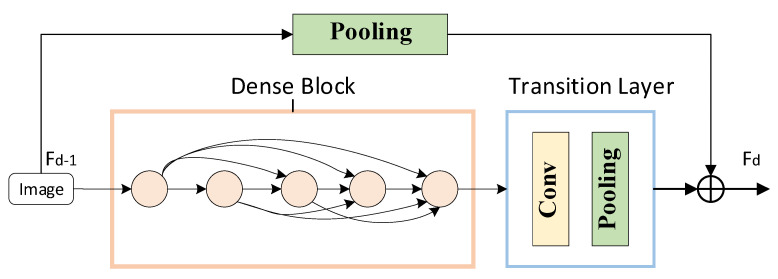
Local Residual Dense Block.

The core unit of LR-DenseNet is LRDB, which consists of a Dense Block, Transition Layer, and residual connection. The LRDB concrete structure is shown in Figure 3. Its own input information transfer from the front to rear makes a convolution of each subsequent layer before it can get the characteristics of the memory and realize the continuous memory mechanism. This kind of structure has a positive influence on the characteristics of the transmission, and makes the neural network have a continuous memory between each layer.

(3) Fusion fault feature abstraction

The input data in this paper is an energy spectrum feature map that considers the complexity and characteristic difference difficult. By adding the fault feature abstraction module in the network, which consists of a global average pooling layer, it reduces the complexity of the network model to some extent and can further extract the characteristics of abstract processing, processing only that which needs to be retained for classification of abstract characteristics. It is suitable for the classification of energy spectrum characteristic map.

**Figure 4:**
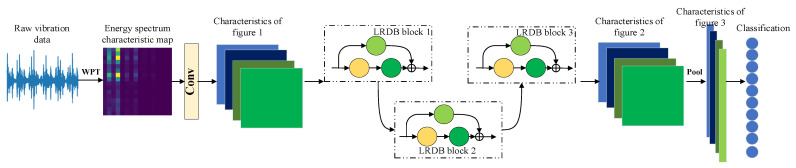
LR-DenseNet Intelligent Fault Diagnosis Model.

In this paper, a Lightweight Dense Residual Network fault diagnosis model LR-DenseNet is constructed based on the LRDB and energy spectrum matrix. The model framework is shown in Figure 4. 

Specific network architecture parameters are shown in Table 3.

Meanwhile, the specific training process of the LR-DenseNet model designed in this paper is mainly divided into three steps: data acquisition and processing, network model training, and online fault diagnosis using the trained model. The details are summarized as follows:

(1) Data acquisition and processing

The vibration sensor is used to collect the original vibration data, which is transmitted to the industrial computer equipped with Win7 system through PCI board card, and the industrial computer makes the mat format data set. Each sample in the data set is numbered to obtain the sample sequence {x,L}, which represents the fault type of L corresponding to the timing signal x.

Information processing operations are performed based on mat dataset samples. Firstly, the 2^8^ branch signals after reconstruction are obtained by wavelet packet transform, and the energy information contained in them is calculated. The energy spectrum feature map is obtained by processing the energy information, and the data sample set {F,L} is made for subsequent network input. Finally, the obtained energy spectrum feature map sample set {F,L} is divided into data sets.

(2) Network training

The LR-DenseNet intelligent fault diagnosis network model was built and includes three parts: shallow feature extraction, novel local residual dense block, and dense fault feature abstraction. At the same time, the model parameters were initialized, and the iteration times and training batches were set through experiments. The LR-DenseNet model was trained on the basis of the constructed sample dataset. During the training process, the error loss function was used to calculate the training error of the model. Then, based on the loss error, the parameters in the model were iteratively updated using the backpropagation mechanism until the model converges, indicating that the model has been trained. At the same time, the validation set is used to verify the model parameters in the optimization process, and the optimal model is saved through the program in the verification process. Finally, the diagnostic performance of the model is tested using the test set.

(3) Online diagnosis

The first two steps belong to off-line model training, and then the trained model is used to diagnose bearing faults online. The model built in this paper has a built-in fault prediction port. After the vibration data samples to be predicted are sent to the network model, the fault port can diagnose the fault conditions of the samples in real time and give an early warning according to the fault conditions.

### 3.3. Adaptive Fault Detection Method Based on Transfer Learning

The model combining an energy spectrum feature map and LR-DenseNet proposed in the previous chapter found that the classification accuracy of some categories was slightly poor when using a confusion matrix for analysis, and the training time of the model was long. This chapter will improve the above problems. Firstly, a transfer learning strategy is introduced to shorten the training time through cross-domain transfer learning. At the same time, an improved feature fusion layer based on the channel attention module is creatively proposed to improve the classification accuracy of the model. The improved model is the LR-DenseNet model designed in this chapter. After a series of experiments, it is found that the LR-DenseNet model has a great improvement in the recognition accuracy. The training time is greatly reduced, the model can achieve convergence faster, and the reliability and robustness of the model are greatly improved. The LR-DenseNet intelligent fault detection model proposed in this paper is a new lightweight CNN architecture that integrates residual learning and dense connection ideas. It strengthens the information flow between each layer of the deep network model, makes the transfer of features and gradients more effective, and alleviates the problem of vanishing gradients. At the same time, based on maintaining superior diagnostic accuracy, the number of parameters, and computational complexity of the model are greatly reduced.

**Figure 5:**
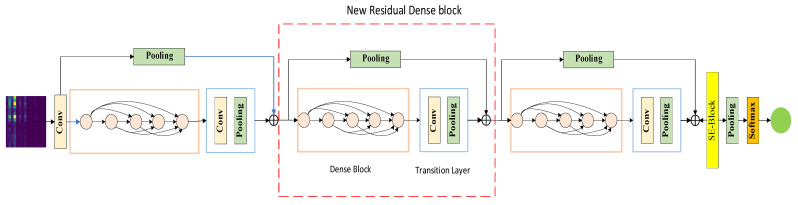
LR-DenseSENet Model.

In this paper, a feature fusion mechanism based on locally dense residual block and channel attention mechanism is designed. According to the importance of different channel features, different weights are allocated to realize the suppression of low efficiency features and the full use of high efficiency features, which greatly improves the network’s feature processing strategy. As an embedded module, the channel domain attention module will be placed in two places by scholars in most cases: one is at each feature extraction layer of the network, and the other is at the output layer. These two have the best effect. To ensure the efficiency of the designed model and make it more lightweight, this paper chooses the latter of the two positions. Se-block is placed before the fully connected layer of the model, which will not increase the amount of calculation too much. The model performance is improved to a certain extent. The improved LR-DenseSENet network model based on channel domain attention mechanism is shown in Figure 5.

**Figure 6:**
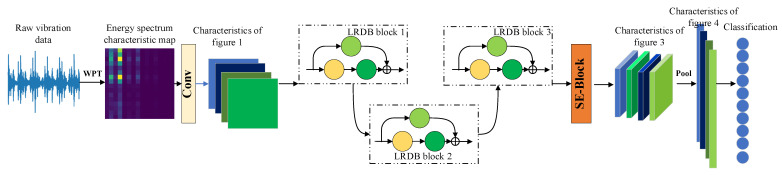
LR-DenseSENet Adaptive Fault Diagnosis Model.

In this paper, channel attention is embedded into the network based on the LRDB and energy spectrum matrix, and then a lightweight dense residual adaptive fault diagnosis model LR-DenseSENet is constructed. The schematic diagram of the model framework is shown in Figure 6. 

Specific network architecture parameters are shown in Table 4.

As can be seen from the figure, it is mainly composed of the LRDB module and SE-block module stacked. The general working process of the model is divided into the following steps: data acquisition and processing, network training, and online diagnosis. Network training is different from LR-DenseNet, as follows:

The LR-DenseSENet intelligent fault diagnosis network model was built, including four parts: shallow feature extraction, new local residual dense block, dense fault feature abstraction, and channel feature fusion. At the same time, the model parameters were initialized, and the iteration times and training batches were set through experiments. The specific architecture of the model is shown in Table 4.

The model processing process is mainly as follows: First, the input energy spectrum feature map is processed, and 16 3 × 3 convolution checks are used for preliminary convolution processing. Then, the feature map obtained after processing is fed into the local residual-dense module LRDB. The number of convolution kernels of the three LRDB blocks is 16, 32, and 64, and the output feature map size of the three modules is 32 × 32, 16 × 16, and 8 × 8, respectively. Both of them use the way of padding = same to make the size of the feature map before and after the processing consistent. After the stitching, the transition layer is used to compress the feature, which is convenient for further processing. Then, the SE module is used to implement the channel domain attention mechanism to weigh the feature channels with different importance degrees.

Finally, the global mean pooling layer is used to further process the feature map, and the feature vectors used to characterize the input feature information are obtained. Based on these feature vectors, the classifier is used to classify and diagnose the feature map.

The LR-DenseSENet model was trained based on the constructed sample dataset. The error loss function was used to calculate the training error of the model in the training process, and then the parameters in the model were iteratively updated based on the loss error using the backpropagation mechanism until the model converges, which means that the model has been trained. At the same time, the validation set is used to verify the model parameters in the optimization process, and the optimal model is saved through the program in the verification process. Finally, the diagnostic performance of the model is tested using the test set.

## 4. Experimental Analysis

### 4.1. Model and Training Process Parameter Settings

To verify the superiority of the proposed model, a sample dataset of energy spectrum characteristic graph was used for validation experiments. The dataset was divided into five sub-datasets according to load, and each sub-dataset represented a load, namely 0HP, 1HP, 2HP, 3HP, and variable load. Each situation was divided into 10 categories, and the data set was divided in a ratio of 7:3 to obtain the training set and test set for subsequent experiments, with 1260 training set pictures and 540 test set pictures in each category. The initialization of relevant parameters of the model is shown in Table 5.

### 4.2. Feature Extraction Effect

**Figure 7:**
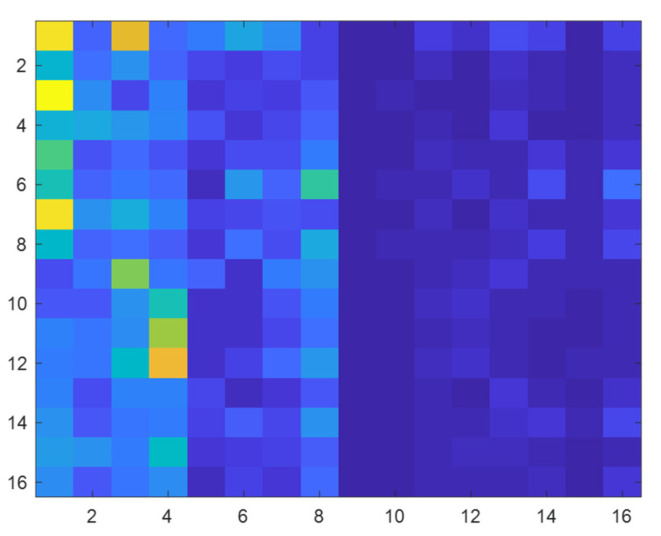
Normal state energy spectrum matrix.

**Figure 8:**
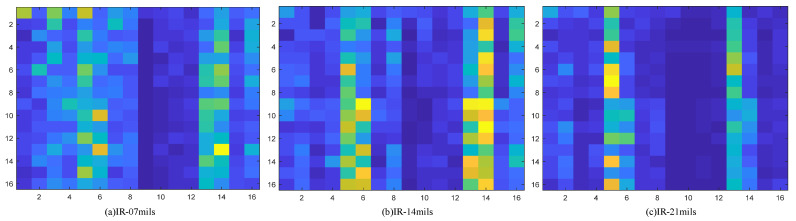
Inner ring fault corresponding to energy spectrum matrix, where (**a**) represents the type of fault with 7 mils in the inner ring, (**b**) represents the type of fault with 14 mils in the inner ring, and (**c**) represents the type of fault with 21 mils in the inner ring.

**Figure 9:**
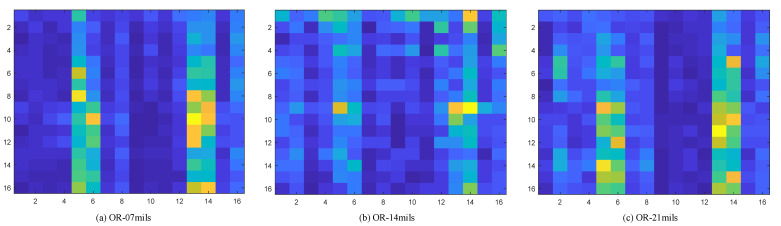
Outer ring fault corresponding to energy spectrum matrix, where (**a**) represents the type of fault with 7 mils in the outer ring, (**b**) represents the type of fault with 14 mils in the outer ring, and (**c**) represents the type of fault with 21 mils in the outer ring.

**Figure 10:**
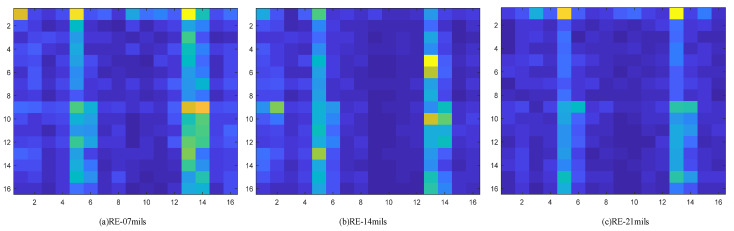
Corresponding energy spectrum matrix of rolling element fault, where (**a**) represents the type of fault with 7 mils in the rolling element, (**b**) represents the type of fault with 14 mils in the rolling element, and (**c**) represents the type of fault with 21 mils in the rolling element.

To verify the effectiveness of the energy spectrum feature graph as a fault feature representation method, a verification experiment was designed; the experiment is based on the environmental load of 2 HP and selects four health condition of bearing vibration data wavelet packet transform. Based on the results of the transformation of the energy spectrum characteristic figure of building, the building is completed on the distribution of energy using histogram visualization, which at the same time draws the corresponding energy spectrum feature maps. The feature extraction effect is shown in the Figure 7, Figure 8, Figure 9 and Figure 10.

Through the experiment and the argument, it can be concluded that the proposed new 2d time-frequency characteristic expression method based on wavelet packet time-frequency transform can fully extract the fault signal characteristic information and a more detailed analysis of the signal at the same time, due to its ability to fully extract and represent the characteristics of the fault information and make various characteristics between the fault type more distinct. Compared with other feature extraction methods, the proposed method can accelerate the convergence speed of subsequent diagnosis models to a certain extent. Therefore, the energy spectrum feature graph is adopted as the expression form of fault features and is used as the input of subsequent networks.

### 4.3. Recognition Accuracy and Performance Analysis of LR-DenseNet Model

**Figure 11:**
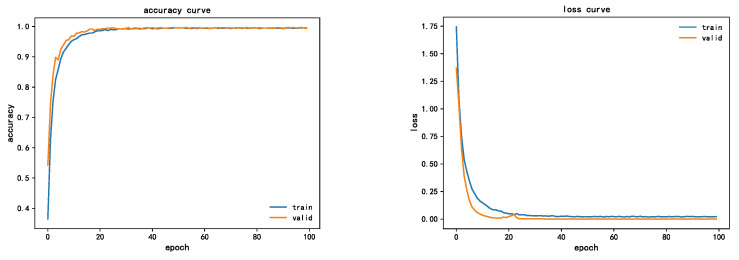
LR-DenseNet model accuracy and loss error curve.

CWRU dataset [35] was selected for verification in this experiment. This dataset is divided into four sub-datasets according to the load condition, and each sub-dataset represents a load, which is 0HP, 1HP, 2HP, and 3HP, respectively. Each case corresponds to four fault modes: normal, inner ring fault, outer ring fault, and rolling body fault, among which three fault modes: inner ring fault, outer ring fault, and rolling body fault correspond to three fault degrees: 7MILS, 14MILS, and 21MILs, respectively. In summary, one health state and three defect states, each with three severe damage degrees, constitute the dataset of energy spectral features used in the experimental validation. There were 10 fault types and 18000 groups of sample data, 70% of which were used to train the network model, and the rest (30%) were used to test network performance. The training batch of this experiment was set as eight. At the same time, the initial learning rate was set to 0.001, the Adam optimizer was used for adaptive adjustment, and 100 rounds of iterative training were carried out. Figure 11 shows the change curve of accuracy and loss error of the LR-DenseNet model, wherein the bearing load is 2Hp.

As can be seen from the figure, after 40 iterations of convergence, the accuracy of the training set finally converges to 99.32%, and the accuracy of the verification set finally converges to 94.25%, which has a good effect. From the loss error convergence curve of the model, the training set and verification set converge to 0.02, basically close to 0. It can be seen from both accuracy curve and loss value curve that the training curve and test curve gradually tighten with little fluctuation, indicating that the network structure is a good model.

### 4.4. Attention Mechanism and Transfer Learning Effect Analysis

**Figure 12:**
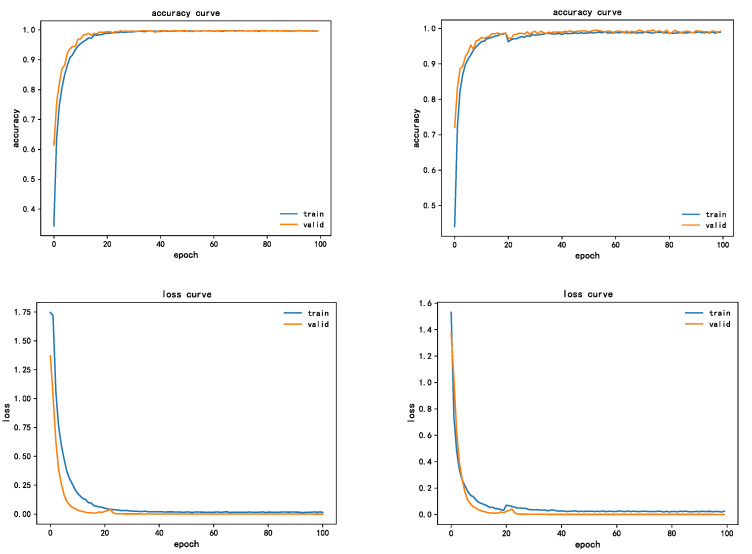
Comparison of the performance of the two attention mechanisms.

To verify the suitability of channel attention mechanism with the design model of this project, a comparative experiment was conducted between the channel attention mechanism and spatial attention mechanism. In the experiment, 150 Epochs were trained, and energy spectrum characteristic data set was selected. The model for comparison is Spatial Attention (SPA) [36], which is the same as the Attention steps of SE-Net and is also divided into three steps: channel compression, channel transformation, and attention weighting. The comparison of the SE attention module (left picture) and SPA attention module (right picture) is shown in Figure 12.

It is not difficult to see from the figure that the model with SE attention mechanism was able to achieve convergence within five epochs with fast convergence speed and a smooth curve, and the diagnostic accuracy was 100%, whereas the model with SPA attention mechanism was worse, and the convergence was achieved after 20 epochs, and the convergence process had obvious vibration. Finally, the accuracy of the validation set did not reach 100%. By contrast, the convergence performance of the model was improved by 15% with the addition of the SE attention mechanism, the accuracy was higher, and the rate of error convergence was relatively fast, which verified the superiority of the SE attention mechanism in this paper.

**Figure 13:**
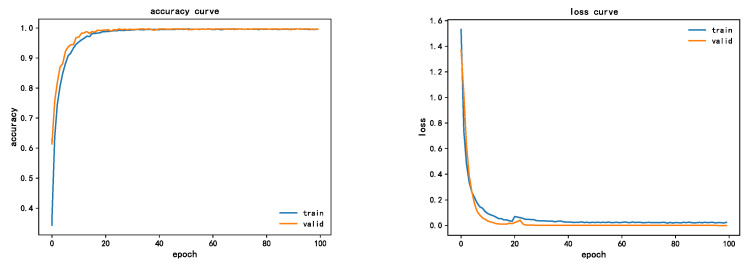
LR-DenseSENet model training accuracy and error curve.

To verify the performance advantage of using transfer learning in this paper, the model involved in this paper is evaluated from two aspects of training accuracy and training duration. The fault diagnosis accuracy of the model with the addition of transfer learning and SE module is shown in Figure 13.

As can be seen from the figure, from the perspective of convergence performance, the improved model will converge in 20 epochs. In terms of diagnosis accuracy, the final diagnosis accuracy reaches 99.59%, which can identify the fault type well. From the error loss curve, the convergence speed is fast, and the final error is less than 0.05, with a good loss error, which indicates that transfer learning and SE attention mechanism have a good performance improvement for the model design.

Then, the performance advantages brought by transfer learning are demonstrated by combining training duration and accuracy. Table 6 shows the comparison of experimental results.

It can be seen that in the same training duration, the accuracy of the model with transfer learning is slightly higher, whereas the training duration of the model without transfer learning is usually more than 500 min, and only 400 min after transfer learning is added. In general, the application of transfer learning can greatly shorten the training time and achieve high accuracy, which proves that the introduction of transfer learning brings good effect to the model.

### 4.5. Overall Performance Analysis of the Model

Then, the overall performance of the LR-DenseSENet model based on transfer learning is analyzed, and three experiments are used to discuss the advantages of the model’s overall performance from three aspects.

Firstly, to further verify the superiority of the fault diagnosis model based on the combination of energy spectrum matrix and LR-DenseSENet model, the performance of the proposed method is compared with the commonly used feature extraction algorithms at the present stage. WPT is the wavelet packet transform method used in this paper, and FFT represents the time-frequency graph obtained by Fourier transform. Two-dimensional Gray Pixel Images are two-dimensional grayscale images [37], which are 64 × 64 two-dimensional image data formed by normalizing the amplitude of the original vibration signal to 0–255. The comparison results are shown in Table 7.

It can be seen from the table that the feature representation method based on the two-dimensional gray map has the lowest accuracy, only 93.7% on the training set and 92.8% on the test set. The feature representation method based on Fourier transform spectrum has the accuracy of 95.6% on the training set and 94.6% on the test set. As a feature representation method, the energy spectrum feature based on wavelet packet transform in this paper has the best effect, and its accuracy in the training set and test set of energy spectrum feature map is more than 95%, and the accuracy of diagnosis based on LR-DenseSENet model on the training set is 99.59%. At the same time, the accuracy rate on the test set reaches 98.7%. These data further demonstrate the effectiveness of using the energy spectrum characteristic map as the fault feature representation method in this paper and prove the performance advantage of the adaptive fault diagnosis model based on the combination of the energy spectrum characteristic map and the LR-DenseSENet model.

Next, we compare the lightweight model in this paper with several common deep learning models in terms of effect and number of parameters. MATLAB is used to realize the last three network architectures. The effect pairs are shown in Table 8, where FLOP (Floating point Operations) represents the floating-point operand of the specified model. Its value reflects the complexity of the model. No.of Parameters refers to the Parameters in the network model; Accuracy refers to the accuracy of training results.

**Figure 14:**
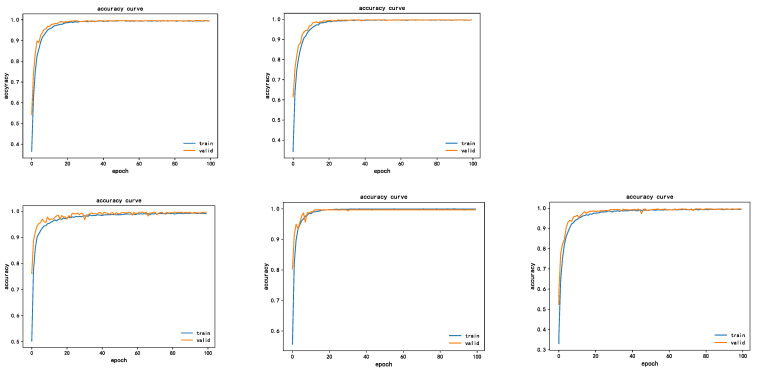
Accuracy of different deep learning models.

The effect pairs are shown in Figure 14. Experimental results show that the number of parameters of the lightweight LR-DenseSENet model designed in this paper is only 0.75 M, only 1/5 of that of VGG-19 model, and the number of parameters is similar to that of other deep models. From the perspective of model complexity, it is only 71.3 M, lower than ResNet-56’s 90 M. It is even lower than 250 M of VGG-19, but it has achieved 99% accuracy. The comparison results show that the model designed in this paper has achieved 99.4% accuracy under the condition of fewer parameters and lowest model complexity, which has significant diagnostic performance.

**Figure 15:**
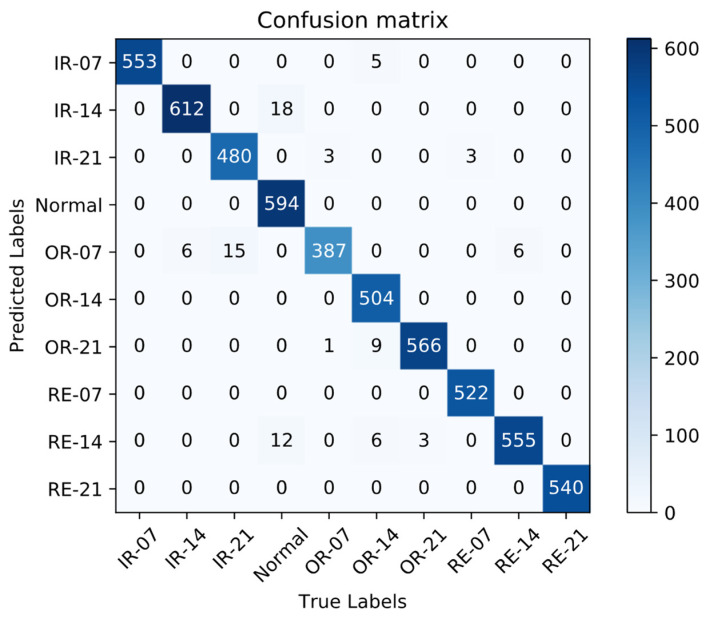
Confusion Matrix Classification Visualization.

To further demonstrate the recognition effect of LR-DenseNet for different fault types, the Confusion matrix [38] is used to further express the model recognition effect visually. The test result shown in Figure 15 is the Confusion matrix, where the horizontal axis represents the real fault label, the vertical axis represents the network prediction of labels, the diagonal axis realizes the correct classification of the number of samples, and the rest of the grid in this picture represents the number of sample classification errors, which at the same time can be understood according to the specific mistake in the horizontal ordinate that determines the kind of fault type.

As can be seen from the figure, the LR-DenseSENet model has a good ability to distinguish fault categories and accurately locate faults with a small number of errors. The performance of the LR-DenseSENet model is better than that of LR-DenseNet, which proves the effectiveness and strong learning ability of the model.

### 4.6. Model Robustness Analysis under Variable Load Environment

To verify the robustness and generalization ability of the LR-DenseSENet model for bearing fault diagnosis, five data sets were made, in which the sample data of 0HP, 1HP, 2HP, and 4HP were made into four data sets A, B, C and D, respectively, and then the sample data of B, C, and D were combined and split. Data set E is the variable load data set. Training is conducted to observe the adaptability of the model under different load environments. The training results are shown in Table 9.

It can be concluded from the experimental results that the LR-DenseSENet model has good accuracy under different load environments, can effectively identify the fault type, and can converge to the minimum error. Meanwhile, after adding variable load data set E, the LR-DenseSENet model can still converge, and the accuracy is close to 99%. The loss error is close to 0, which further proves the robustness and generalization ability of the LR-DenseSENet model for bearing fault diagnosis.

### 4.7. On-Line Diagnosis

**Figure 16:**
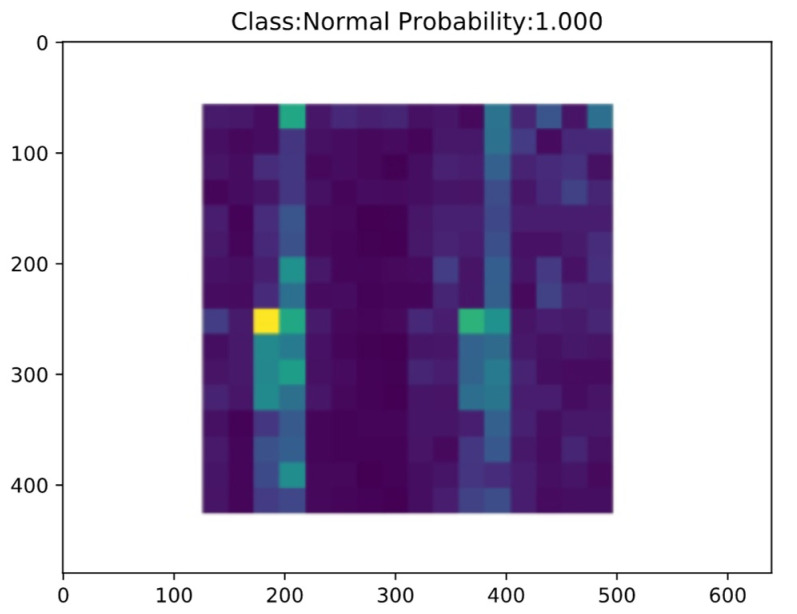
Bearing normal condition online diagnosis results.

**Figure 17:**
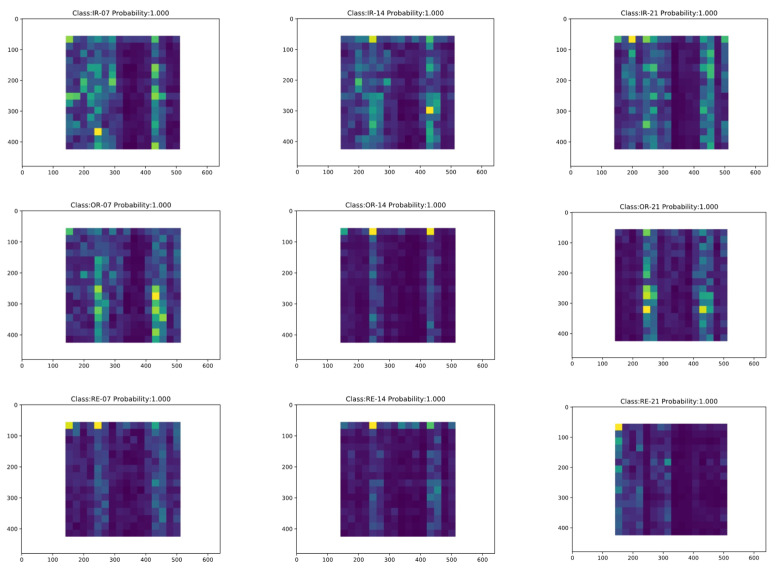
LR-DenseNet model online diagnosis results.

Firstly, the offline training phase of LR-DenseSENet model was carried out, and then the online diagnosis task of fault type was carried out [39]. The trained LR-DenseSENet model is saved, and then the online diagnosis port is used to diagnose the state categories of feature images in the test set of the divided energy spectrum feature graph by using the designed model, to realize the online diagnosis of faults [40]. After the input data, the online prediction results of LR-DenseSENet model are shown in Figure 16 and Figure 17.

Figure 16 shows the online diagnosis results of the Normal fault type samples of rolling bearings, where NL stands for Normal, i.e., Normal fault type, and Probability and the following number represent the accuracy of prediction. The model can better identify the Normal fault type. The sample diagnosis results of the other nine fault types are shown in Figure 17.

The fault classification involved mainly includes three main fault modes: inner ring fault, outer ring fault, and rolling body fault, among which each fault mode has three faults with different damage degrees, and a total of nine fault types. When online bearing fault prediction is carried out, the energy spectrum images in the test set are firstly input into the prediction system, and the system uses the trained network model to make an online diagnosis, and outputs the diagnosis results and predicted probability. It can be seen from Figure 15 and Figure 16 that the online diagnosis module has a good diagnosis effect, and the identification accuracy of all fault categories reaches over 99%. The LR-DenseSENet model can achieve a good effect overall, and the model can extract and learn the characteristics of samples well. Therefore, the LR-DenseSENet model can be used for predicting with high accuracy.

## 5. Conclusions

As the core component of power equipment, rolling bearing has an important influence on the safe operation of the smart grid. Therefore, it is of great significance to diagnose the fault. In this paper, the rolling bearing is selected as the research object, the energy spectrum feature map is proposed as a novel fault feature representation method, a lightweight LR-DenseSENet adaptive fault diagnosis model is designed for diagnosis and classification, and the experiment is carried out based on the CWRU dataset, the standard data set of motor bearing fault diagnosis. Thus, the efficiency and generalization of the LR-DenseSENet model are further proved.

## Figures and Tables

**Table 1:** Methods based on fault detection.

Refs.	Algorithms	Core Ideas	Achieved Lightweight	Implemented Cross-Domain Transfer Learning and Feature Fusion
[32]	MSMFCNN, multi-scale multi-sensor feature fusion convolutional neural network	It fused the information provided by multiple sensors and conducted fault diagnosis based on CNN.	No	No
[33]	an enhanced deep residual network with multilevel correlation information	It is used to process the feature information obtained by wavelet packet transformation.	No	No
[34]	a fault diagnosis method based on IMFs and WDenseNets	The components of vibration signals obtained through empirical mode decomposition were weighted and input into WDenseNets for fault identification and classification	No	No
Our method 1.	LR-DenseNet	A Lightweight Dense Residual Network, a new dense residual block, is designed by combining residual connection and dense connection	Yes	No
Our method 2.	LR-DenseSENet	The channel attention and transfer learning are embedded into the network based on LRDB and energy spectrum matrix.	Yes	Yes

**Table 2:** Notation description.

f(t)	The original vibration data signals
$E_{i,j}$	The branch band energy
B	The decomposition coefficient
$S_{i,j}$	The branching signal
$B_{i,j}(n)$	The corresponding wavelet packet coefficient
$e_{i,j}$	The energy value of the corresponding node after normalization
F	The energy spectrum feature vector

**Table 3:** Architectures of proposed LR-DenseNet model.

Layers	Output Size	Details
Convolution	128×128	3×3 Conv. ×64
Avg.Pooling	64×64	2×2 avg.pooling
Dense Block-1	64×64	$\left[\begin{array}{c} 1\times 1\;\mathrm{Conv.}\\ 3\times 3\;\mathrm{Conv.} \end{array}\right]\times 16$
Trans Block-1	32×32	1×1 Conv. ×16 2×2 avg.pooling
Dense Block-2	32×32	$\left[\begin{array}{c} 1\times 1\;\mathrm{Conv.}\\ 3\times 3\;\mathrm{Conv.} \end{array}\right]\times 16$
Trans Block-2	16×16	1×1 Conv. ×16 2×2 avg.pooling
Dense Block-3	16×16	$\left[\begin{array}{c} 1\times 1\;\mathrm{Conv.}\\ 3\times 3\;\mathrm{Conv.} \end{array}\right]\times 16$
Trans Block-3	8×8	1×1 Conv. ×16 2×2 avg.pooling
Classification	1×1	8 × 8 Global avg.pooling, 1000-D fc, softmax

**Table 4:** Architectures of proposed LR-DenseSENet model.

Layers	Output Size	Details
Convolution	128×128	3×3 Conv. ×64
Avg.Pooling	64×64	2×2 avg.pooling
Dense Block-1	64×64	$\left[\begin{array}{c} \mathrm{Conv.}\\ 3\times 3\;\mathrm{Conv.} \end{array}\right]\times 16$
Trans Block-1	32×32	1×1 Conv. ×16 2×2 avg.pooling
Dense Block-2	32×32	$\left[\begin{array}{c} 1\times 1\;\mathrm{Conv.}\\ 3\times 3\;\mathrm{Conv.} \end{array}\right]\times 16$
Trans Block-2	16×16	1×1 Conv. ×16 2×2 avg.pooling
Dense Block-3	16×16	$\left[\begin{array}{c} 1\times 1\;\mathrm{Conv.}\\ 3\times 3\;\mathrm{Conv.} \end{array}\right]\times 16$
Trans Block-3	8×8	1×1 Conv. ×16 2×2 avg.pooling
SE-Block	8×8	Conv.
Classification	1×1	8×8 Global avg.pooling, 1000-D fc, softmax

**Table 5:** Model initialization parameter configuration.

The Parameter Name	LR-DenseNet Setting Network Parameters
Initial value of learning rate	0.0001
The number of iterations	100
Minimum training lot	8
Convolutional zero complement strategy	SAME
Zero pooling policy	SAME

**Table 6:** Comparison of transfer learning effects.

The Model’s Name	Accuracy Rate	Training Duration (min)
LR-DenseNet	93.17%	400
LR-DenseSENet	99.23%	400
LR-DenseSENet based on the TF	99.59%	400

**Table 7:** Comparison of the effects of different two-dimensional feature extraction methods.

A Diagnosis Model	Accuracy of Train	Accuracy of Test
WPT + LR-DenseNet	98.6%	95.1%
WPT + LR-DenseSENet	99.59%	98.7%
FFT+ LR-DenseSENet	95.6%	94.6%

**Table 8:** Comparison of complexity and accuracy of different deep learning models.

Model	FLOPs	No.of Parameters	Accuracy
LR-DenseNet (upper left)	69.1 M	0.61 M	98.6%
LR-DenseSENet (upper right)	71.3 M	0.75 M	99.4%
VGG-19 (lower left)	250 M	5.00 M	97%
ResNet-56 (lower middle)	90 M	0.88 M	99.5%
DenseNet-40 (lower right)	190 M	0.79 M	99.1%

**Table 9:** Comparison of model training performance of failure mode determination layer under four loads.

The Data Set	Number of CONVERGENCES	The Training Set		The Test Set	
		Error Loss	Accuracy Rate	Error Loss	Accuracy Rate
A	48	0.005	99.65%	0.006	99.46%
B	38	0.010	99.66%	0.011	99.01%
C	40	0.009	100%	0.003	99.95%
D	46	0.012	99.52%	0.004	99.56%
E	50	0.015	98.75%	0.018	98.05%

## Data Availability

The data provided by this study is available on Bearing Data Center Official Website of Case Western Reserve University. Available online: https://csegroups.case.edu/bearingdatacenter/ home, accessed on 10 April 2022.

## References

[1] Zhang X., Zhao B.Y., Lin Y. (2021). Machine Learning Based Bearing Fault Diagnosis Using the Case Western Reserve University Data: A Review. IEEE Access.

[2] Guo T.T., Zhang T.P., Lim E., Lopez-Benitez M., Ma F., Yu L.M. (2022). A Review of Wavelet Analysis and Its Applications: Challenges and Opportunities. IEEE Access.

[3] Taubenschuss U., Santolik O. (2019). Wave Polarization Analyzed by Singular Value Decomposition of the Spectral Matrix in the Presence of Noise. Surv. Geophys..

[4] Gao J.P., Wang X., Wu R.W., Xu X. (2021). A New Modulation Recognition Method Based on Flying Fish Swarm Algorithm. IEEE Access.

[5] de Sena A.P.C., de Freitas I.S., Lima A.C., Sobrinho C.A.N. (2021). Fuzzy diagnostics for gearbox failures based on induction motor current and wavelet entropy. J. Braz. Soc. Mech. Sci. Eng..

[6] Xue Y.J., Cao J.X., Wang X.J., Li Y.X., Du J. (2019). Recent Developments in Local Wave Decomposition Methods for Understanding Seismic Data: Application to Seismic Interpretation. Surv. Geophys..

[7] Ji J.J., Qu J.F., Chai Y., Zhou Y.M., Tang Q., Ren H. (2018). An algorithm for sensor fault diagnosis with EEMD-SVM. Trans. Inst. Meas. Control.

[8] Kim J., Kang M., Jeong I.K., Jun H., Kim J.M., Choi B.K. Real-Time and Energy-Efficient Bearing Fault Diagnosis Using Discriminative Wavelet-Based Fault Features on a Multi-Core System. Proceedings of the IEEE Intemational Conference on Prognostics and Health Management.

[9] Pang B., Tang G.J., Zhou C., Tian T. (2018). Rotor Fault Diagnosis Based on Characteristic Frequency Band Energy Entropy and Support Vector Machine. Entropy.

[10] Vargas-Hakim G.A., Mezura-Montes E., Acosta-Mesa H.G. (2022). A Review on Convolutional Neural Network Encodings for Neuroevolution. IEEE Trans. Evol. Comput..

[11] Sohn I. (2021). Deep belief network based intrusion detection techniques: A survey. Expert Syst. Appl..

[12] Liu Y., Xia X.G., Zhang Z.Q., Zhang H.L. (2017). Distributed Space-Time Coding Based on the Self-Coding of RLI for Full-Duplex Two-Way Relay Cooperative Networks. IEEE Trans. Signal Process..

[13] Yu Y., Si X.S., Hu C.H., Zhang J.X. (2019). A Review of Recurrent Neural Networks: LSTM Cells and Network Architectures. Neural Comput..

[14] Song C.H., Han G.J., Zeng P. (2022). Cloud Computing Based Demand Response Management Using Deep Reinforcement Learning. IEEE Trans. Cloud Comput..

[15] Yuwono M., Qin Y., Zhou J., Guo Y., Celler B.G., Su S.W. (2016). Automatic bearing fault diagnosis using particle swarm clustering and Hidden Markov Model. Eng. Appl. Artif. Intell..

[16] Tamilselvan P., Wang P.F. (2013). Failure diagnosis using deep belief learning based health state classification. Reliab. Eng. Syst. Saf..

[17] Mark W.D. (2015). Time-synchronous-averaging of gear-meshing-vibration transducer responses for elimination of harmonic contributions from the mating gear and the gear pair. Mech. Syst. Signal Proc..

[18] Wang B.X., Ding C.C. (2020). Hierarchical Frequency-Domain Sparsity-Based Algorithm for Fault Feature Extraction of Rolling Bearings. IEEE Trans. Instrum. Meas..

[19] Wang S.B., Chen X.F., Tong C.W., Zhao Z.B. (2017). Matching Synchrosqueezing Wavelet Transform and Application to Aeroengine Vibration Monitoring. IEEE Trans. Instrum. Meas..

[20] Barbosh M., Singh P., Sadhu A. (2020). Empirical mode decomposition and its variants: A review with applications in structural health monitoring. Smart Mater. Struct..

[21] Li H.B., Wu Z.H., Xue K.X., Yang G.A. Research on Aero-engine Bearing Fault Using Acoustic Emission Technique Based on Wavelet Packet Decomposition and Support Vector Machine. Proceedings of the 2nd IEEE Advanced Information Technology, Electronic and Automation Control Conference (IAEAC).

[22] Wu C.H., Yue J.G., Wang L., Lyu F. (2019). Detection and Classification of Recessive Weakness in Superbuck Converter Based on WPD-PCA and Probabilistic Neural Network. Electronics.

[23] Song C.H., Sun Y.Y., Han G.J., Rodrigues J. (2021). Intrusion detection based on hybrid classifiers for smart grid. Comput. Electr. Eng..

[24] Ma M., Sun C., Chen X.F. (2017). Discriminative Deep Belief Networks with Ant Colony Optimization for Health Status Assessment of Machine. IEEE Trans. Instrum. Meas..

[25] Yin Z., Hou J. (2016). Recent advances on SVM based fault diagnosis and process monitoring in complicated industrial processes. Neurocomputing.

[26] Tabrizi A., Garibaldi L., Fasana A., Marchesiello S. (2015). Early damage detection of roller bearings using wavelet packet decomposition, ensemble empirical mode decomposition and support vector machine. Meccanica.

[27] Hu Z.K., Gui W.H., Yang C.H., Deng P.C., Ding S.X. (2011). Fault Classification Method for Inverter Based on Hybrid Support Vector Machines and Wavelet Analysis. Int. J. Control Autom. Syst..

[28] Tan R., Zhang Y., Zheng T.X., Yang B., Wang Y.J. Valve clearance fault diagnosis of an internal combustion engine based on wavelet packets and k-nearest neighbors. Proceedings of the International Conference on Civil, Architecture and Environmental Engineering (ICCAE).

[29] Li T., Zhao Z., Sun C., Yan R., Chen X. (2020). Multi-scale CNN for Multi-sensor Feature Fusion in Helical Gear Fault Detection. Procedia Manuf..

[30] Xiong S., He S., Xuan J., Xia Q., Shi T. (2020). Enhanced deep residual network with multilevel correlation information for fault diagnosis of rotating machinery. J. Vib. Control.

[31] Chong S., Yan R., Hesheng T., Romeo M.L. (2021). A fault diagnosis method for an electro-hydraulic directional valve based on intrinsic mode functions and weighted densely connected convolutional networks. Meas. Sci. Technol..

[32] Song C., Liu S., Han G., Zeng P., Yu H., Zheng Q. (2022). Edge intelligence based condition monitoring of beam pumping units under heavy noise in the industrial Internet of Things for Industry 4.0. IEEE Internet Things J..

[33] Fausing Olesen J., Shaker H.R. (2020). Predictive Maintenance for Pump Systems and Thermal Power Plants: State-of-the-Art Review, Trends and Challenges. Sensors.

[34] Liu C.C., Zhuo F., Wang F. (2021). Fault Diagnosis of Commutation Failure Using Wavelet Transform and Wavelet Neural Network in HVDC Transmission System. IEEE Trans. Instrum. Meas..

[35] Li Z.M., Wang X.H., Yang R. Fault Diagnosis of Bearings Under Different Working Conditions based on MMD-GAN. Proceedings of the 33rd Chinese Control and Decision Conference (CCDC).

[36] Woo S.H., Park J., Lee J.Y., Kweon I.S. CBAM: Convolutional Block Attention Module. Proceedings of the 15th European Conference on Computer Vision (ECCV).

[37] Lee C., Lee C., Kim C.S. (2013). Contrast Enhancement Based on Layered Difference Representation of 2D Histograms. IEEE Trans. Image Process..

[38] Simon D., Simon D.L. (2010). Analytic Confusion Matrix Bounds for Fault Detection and Isolation Using a Sum-of-Squared-Residuals Approach. IEEE Trans. Reliab..

[39] Song C.H., Xu W.X., Han G.J., Zeng P., Wang Z.F., Yu S.M. (2021). A Cloud Edge Collaborative Intelligence Method of Insulator String Defect Detection for Power IIoT. IEEE Internet Things J..

[40] Wu Z.H., Jiang H.K., Liu S.W., Zhao K. (2021). A deep ensemble dense convolutional neural network for rolling bearing fault diagnosis. Meas. Sci. Technol..

